# Progress and remaining priorities in geospatial analysis of health inequalities

**DOI:** 10.1038/s43856-026-01993-w

**Published:** 2026-11-03

**Authors:** Peter M. Macharia, Nancy Fullman, Ezra Gayawan

**Affiliations:** 1 Department of Public Health, Institute of Tropical Medicine, Antwerp, Belgium; 2 Population & Health Impact Surveillance Group, Kenya Medical Research Institute-Wellcome Trust Research Programme, Nairobi, Kenya; 3 Department of Global Health, University of Washington, Seattle, WA USA; 4 Department of Statistics, Federal University of Technology, Akure, Nigeria

**Keywords:** Epidemiology, Epidemiology

## Abstract

Health outcomes, services and their drivers can now be mapped at fine spatial and temporal resolution, driven by growth in data, computing power and geospatial methods. Here we discuss what the field has achieved, where estimates can outpace their use, and what actions could close the gap.

Health outcomes vary from place to place, yet national averages can mask this. A country on track to meet coverage targets frequently contains areas where large populations remain unreached. Making these variations visible and usable is the work of spatial epidemiology, a field at the intersection of health and geography^1,2^. Over recent decades, the field has been reshaped by wide access to georeferenced household surveys, geocoded health facility lists and satellite imagery, alongside advances in spatial and spatiotemporal statistics^3^, computing power and, more recently, geospatial artificial intelligence (GeoAI). We mainly focus on low- and middle-income countries (LMICs), where the burden of disease is highest and inequalities within countries are widest.

The field’s clearest achievement has been high-resolution mapping of disease burden, intervention coverage and other health outcomes, reflecting a shift from describing where disease occurs to generating predictions where no data were collected. These continuous estimates can expose local stagnation that national progress can conceal. For instance, continental modelling showed where malaria transmission fell as control scaled up and where it did not^4^. Small-area estimation of under-five mortality across 35 African countries revealed which subregions lagged furthest behind national progress, and which areas were catching up^5^. In Kenya, combining environmental suitability modelling with geostatistical mapping found only 4% of the landmass suitable for podoconiosis, with more than 60% of cases in just three of 47 counties, a pattern that favours locally targeted interventions over nationally uniform programmes^6^. Beyond mapping levels, space-time models now track how patterns shift and where new hotspots emerge, and link disease to climate, population and environment; they are increasingly used to inform elimination efforts and service planning. Much of this work depends on open, high-resolution gridded population data, which provide the denominators and the population counts against which nearly every estimate is measured, though their accuracy varies^7^.

Geographic accessibility mapping has advanced in parallel. Global travel-time surfaces now estimate journeys to cities, healthcare, schools and other services, exposing inequalities in who can reach them^8^. More detailed and localised analyses account for traffic congestion, time of day and travel mode, drawing on routing services, vehicle GPS, mobile phone records and crowdsourced road networks that mirror how people actually reach services^9^. Several overlapping vulnerabilities, such as poverty, urban deprivation and travel time to care, now can be examined together and tracked over time through high-resolution gridded estimates of wealth, population and urbanisation, linking access to who is being left behind. In sub-Saharan African cities, residents of informal settlements faced longer walking times to facilities than those in formal areas^10^.

Despite this progress, several weaknesses remain. First, resolution can be mistaken for accuracy. Methods are often used because they are feasible, not because they suit the question at hand; as such, analytic tractability can quickly outrun appropriate data usage, and models at times are pushed further than the data can support. In the Demographic and Health Surveys (DHS), the data source behind most mapping in LMICs, the recorded locations of survey clusters are deliberately shifted to protect confidentiality. Accordingly, the data cannot reliably support estimates below a certain geographic scale, however geospatially fine the model. Changing the unit of analysis can do more than add error; a prevalence estimated at a point, on a grid and across a district are three different quantities, not one quantity measured three ways. Second, the finer the location, the easier it can become to re-identify the people behind an estimate. Third, covariates do not always help; in fact adding them can even make the estimate less certain when data are sparse and clustered, especially, in low-prevalence settings^11^. Fourth, uncertainty is usually calculated but then set aside: decision-makers see the headline estimates but not how uncertain they are, which is vital for judging how far the estimates can be trusted.

There is also a deeper gap. Geospatial estimates are not frequently checked against independent data; a model tested against its own source survey only shows it agrees with itself. Moreover, even robust estimates do not always change decisions. Malaria programme managers in three African countries reported that their main obstacles to using geospatial estimates for decision-making involved interpreting and trusting the maps, not producing more of them^12^.The field now can make maps faster than people have learned to use and act on them.

GeoAI is increasingly pitched as a fix. It can find structure in data that older methods miss, and it helps most where conventional covariates are weakest, such as rapidly changing places. But to date GeoAI primarily changes how the maps are made, not if and how people use them. And GeoAI poses a new risk: when data and methods behind their production become even less visible to end-users, trust can further diminish in parallel^13^.

None of these challenges is new and similar solutions have been offered before: put modellers inside ministries, connect local universities to disease control programmes, fix population denominators on which estimates rely, among others^1^. Nonetheless the field is still grappling with a more philosophical tension: when does finer resolution estimates stop helping decisions and actually start hindering them. For instance, continental-scale mapping initiatives continue to expand^4^ while the underlying data have become precarious^14^; all the while, how exactly geospatial analyses themselves have changed health outcomes remains largely unknown^2^. On its own, adding computing power and model complexity into increasingly weak data foundation is unlikely to produce desired outcomes for the geospatial field. Elevating four key priorities below is therefore vital to catalysing future progress (Table 1).

**Table 1:** Priority areas for geospatial analysis and actions for different actors

Priority	Key challenges	Priority actions to take by actor
**Data**	Routine health data are often incomplete and population figures can be unreliable; not all records are accurately located; datasets are often fragmented across institutions and not always well governed; data from different sources are not routinely combined or cross-checked; conflict-affected, displaced and informal-settlement populations are often under-represented	**Governments and health agencies**: Fund regular censuses and civil registration; set national standards for how geospatial data are collected and shared; maintain an updated register of health facilities; audit data quality; ensure separate systems can share data. **Researchers**: Analyse survey and routine data jointly, and validate estimates against census and registration data where available. **Development partners**: Fund data collection that reaches under-counted groups, and secure infrastructure that allows analysis without revealing individuals’ precise locations
**Open science**	Code and data are not always shared, and written methods alone may not allow results to be reproduced; privacy and ownership concerns often remain unresolved; the work of making research reproducible is not consistently rewarded in careers or funding	**Researchers**: Publish code, documentation and non-sensitive data in open repositories with lasting identifiers, and follow recognised reporting standards. **Universities**: Count open and reproducible work in hiring and promotion, and require deposition of code and data so results are findable, accessible, interoperable and reusable (the FAIR principles). **Funders**: Require data-sharing plans and fund the time and infrastructure to deliver them. **Governments**: Provide secure, controlled access to sensitive data so it can be reused without exposing individuals
**Local capacity**	Modelling expertise is largely concentrated in the Global North, rather than in the LLMICs being mapped; not enough analysts are trained locally, and some who are trained move abroad; funded posts and training programmes are few; computing resources can be limited	**Universities**: Establish local graduate training in spatial analysis and modelling. **Governments**: create modelling posts inside public health institutions, and place analysts within ministries. **Development partners**: Fund training posts, fellowships and long-term mentorship; back regional centres of excellence led locally rather than from abroad; support access to computing; and let local analysts lead major modelling investments. **Professional societies**: Build mentorship networks and communities of practice across institutions and countries.
**Evaluation**	Targeted geospatial programmes are not systematically evaluated for whether they reduced inequality; a change in inequality can be difficult to attribute to the intervention rather than to other causes; researchers and decision-makers often do not exchange feedback; models are often validated against their own source data rather than independent data	**Researchers**: Test estimates against independent data; report whether an estimate changed a decision or improved equity; communicate uncertainty in a form decision-makers can use. **Governments and policymakers:** Create the mandate and funding for evaluation; use existing review processes, for lighter evaluation; frame evaluation as programme improvement rather than audit. **Programme managers**: Apply estimates in planning and report back to researchers on what worked. **Funders**: Support evaluation of real-world impact, including rapid and lighter approaches, not only the development of new models.

The first priority is data, which sets the limit of what can be mapped. Most analyses in LMICs rely on household surveys, routine health records and geocoded lists of facilities. The DHS in particular provides the georeferenced survey locations most mapping depends on. Its brief suspension after the withdrawal of USAID funding was a warning of how fragile that foundation could be^14^; access has been restored, but a source now negotiated country by country can no longer be assumed as a stable, standard input. Routine health records are collected continuously and geolocated by administrative area, nonetheless, they often experience challenges with incompleteness, unreliable population denominators and unclear catchment areas. The gaps disproportionately affect the people already hardest to reach; data on conflict, displacement, remoteness and informal settlements, which mark out the most vulnerable, are among the least used, even as climate change and conflict reshape where people live and where disease risk occurs^15^. These are the people health systems most need to reach, yet they are often the least visible in the same data used to guide that reach. Methods alone cannot fill gaps for measurements never taken.

The second is open science, which would strengthen the validation and reuse of current estimates and methods. Analytic code and modelling inputs like covariate layers and facility datasets are not always shared, and written descriptions alone do not lend themselves to reproduction. Sharing modelling pipelines would raise rigour and save effort, letting people build on existing work instead of recreating the same foundations. Reporting standards already exist, such as Guidelines for Accurate and Transparent Health Estimates Reporting (GATHER) and Spatial Lifecourse Epidemiology Reporting Standards (ISLE-ReSt); stronger incentives for their consistent adoption could help galvanise meaningful change in open science.

The third is capacity: shifting away from a primary focus on methods and moving toward an operating model where local teams and system routinely produce, maintain and use geospatial estimates. Advanced modelling is largely concentrated in institutions in the Global North, even though continental mapping initiatives are mostly focused on LMICs. Such gaps are shaped by unequal access to computational infrastructure and sustained funding for capacity-building. Current incentives reward publication over the slower, longer-term work of embedding these tools into health departments; furthermore staff cannot be expected to learn without designated time and resources^1^. Work that changes practice is built with the people who will ultimately use it; as such strengthening local ownership and interpretation is a critical need going forward^12^.

The fourth priority is evaluation. Studies typically end in recommendations, but whether these recommendations have traction or impact beyond analysis often remains unknown. Regular evaluation does not need to be resource-intensive, especially if approaches such as contribution analysis and rapid evaluation are accepted as credible alternatives to formal impact evaluation. Meaningfully supporting and acting on evaluations can require social capital and political will exceed the financial requirements. Two shifts could help. First, existing country review processes could be leveraged to expand lighter-touch evaluations of geospatial analyses, for example, annual microplanning exercises for routine immunisation programmes. Second, reframing evaluation as a vital input into health system strengthening, resilience and sustainability could lower the sociopolitical stakes for governments, programme managers and funders alike.

Geospatial analysis can now locate health inequalities with precision. What it has yet to do is turn that precision into decisions and programmes that reduces these inequalities. Investment in data, open science, capacity and evaluation is what would move the field beyond producing evidence. Success should be judged not by the resolution of the map, but by whether it changes decisions and, ultimately, outcomes.

## Data Availability

No new data were generated or analysed in this Comment. All sources discussed are cited in the reference list.
